# Is Vitamin D Deficiency Associated with Chronic Lymphocytic Thyroiditis?

**DOI:** 10.14744/SEMB.2021.45202

**Published:** 2021-12-29

**Authors:** Mahmut Kaan Demircioglu, Zeynep Gul Demircioglu, Nurcihan Aygun, Banu Yilmaz Ozguven, Ismail Ethem Akgun, Mehmet Uludag

**Affiliations:** 1. Department of General Surgery, Kars Harakani State Hospital, Kars, Turkey; 2. Department of General Surgery, University of Health Sciences Turkey, Sisli Hamidiye Etfal Training and Research Hospital, Istanbul, Turkey; 3. Department of Pathology, İstanbul Başakşehir Çam and Sakura City Hospital, Istanbul, Turkey

**Keywords:** Lymphocytic thyroiditis, thyroidectomy, Vitamin D deficiency

## Abstract

**Objectives:**

Vitamin D deficiency is a very common global health problem. Evidence from recent studies focuses on the extraskeletal effects of vitamin D (Vit D) deficiency. Chronic lymphocytic thyroiditis (or Hashimoto’s thyroiditis) is an autoimmune disease of the thyroid. Although there are many studies reporting that autoimmune thyroid diseases may be associated with Vitamin D deficiency, this is still a controversial issue that has not yet been proven. In this study, we aimed to appraise whether there is a relationship between lymphocytic thyroiditis diagnosed by histopathological evaluation and Vitamin D deficiency.

**Methods:**

Data of 256 patients whom were operated by a single surgeon in a single center between 2012 and 2017 and whose preoperative vitamin D tests and thyroid pathologies have been collected, were retrospectively evaluated. Due to the pathological examination, two groups were formed considering the presence of lymphocytic thyroiditis (Group 1), and the absence of lymphocytic thyroiditis (Group 2). Vitamin D deficiency was defined as the level <20 ng/mL (50 nmol/L) and Vitamin D insufficiency was defined as the level 21–29 ng/mL (525–725 nmol/L).

**Results:**

There were 108 (92F/16M) patients in Group 1, and 148 (116F/32M) patients in Group 2, and the mean age was lower in Group 1 (p=0.053). The mean vitamin D levels (16.6±15.2 vs. 14±10, p=0.409) and vitamin D deficiency rates (67.6% vs. 72.3%, p=0.416) were found similar between the Groups 1 and 2. No positive significant correlation was found between lymphocytic thyroiditis and vitamin D level or vitamin D deficiency rates. There was a positive correlation between lymphocytic thyroiditis and age, preoperative thyroid-stimulating hormone level, preoperative anti-thyroglobulin ,and anti-thyroid peroxidase levels, but no significant relationship was found with Vitamin D level.

**Conclusion:**

According to our results, lymphocytic thyroiditis was not associated with either Vitamin D deficiency or Vitamin D level.

Vitamin D is considered a steroid hormone rather than a vitamin. Vitamin D regulates the expression of many genes essential for calcium and phosphate metabolism and bone mineralization in human organisms.^[1]^ In recent years, studies on systemic diseases due to vitamin D deficiency and the extraskeletal effects of Vitamin D have gained momentum.^[2]^

Vitamin D3 (or cholecalciferol) is produced by the skin. It is converted to calcitriole 1, 25-hydroxy vitamin D3 levels (1,25[OH]2D3), which is the active form of Vitamin D. Calcitriol (1,25[OH]2D3) is created by hydroxylation in the liver and the action of 1-alpha hydroxylase in the kidney. Calcitriol performs its functions in the cell by binding to the vitamin D receptor (VDR). This binding affects mononuclear phagocytic systems such as monocytes, macrophages, dendritic cells, and immunomodulatory cells such as B and T lymphocytes.^[3]^ In the light of recent studies, it has been shown that lack of Vitamin D may cause extra-skeletal effects. These are autoimmune diseases, metabolic syndromes, cardiovascular diseases, infections, and even some cancers.^[4]^

Recent epidemiological studies have found a significant relationship between the prevalence of autoimmune diseases such as rheumatoid arthritis, multiple sclerosis, systemic lupus erythematosus, type 1 diabetes, and low Vitamin D levels.^[5]^

A correlation was found between low Vitamin D levels and the presence of thyroid peroxidase antibody (TPO-Ab) and gene polymorphism in VDR. Because of this relationship, it has been suggested that it has an effect on autoimmune thyroid diseases.^[3,6]^

Lymphocytic infiltrates are found in the thyroid gland and these infiltrates are typical for the histopathology of autoimmune thyroid diseases.^[7]^ Hashimoto’s thyroiditis and Graves’ disease are the most common autoimmune organ-specific diseases.^[4]^ The pathogenesis of autoimmune thyroiditis is multifactorial and associated with genetic, immune, environmental, and hormonal factors. Genetic predisposing factors can trigger autoimmune thyroiditis by causing loss of self-tolerance as a result of environmental interactions.^[8]^

Chronic lymphocytic thyroiditis (also known as Hashimoto’s thyroiditis) is an autoimmune disease that typically originates from T-cells, is histopathologically characterized by intrathyroidal infiltration, thyrocyte destruction and hypothyroidism, and affects B and T lymphocytes, dominated by the CD4+ T helper 1 subtype.^[9]^

In Graves’ disease, lymphocyte infiltration is not severe and mainly CD4 + T helper 2 cells take the lead.^[4]^ Although there are many studies reporting that autoimmune thyroid diseases may be associated with Vitamin D deficiency, there are also studies reporting that there is no relationship, and this is still a controversial issue.^[1,6,10]^ In studies evaluating the correlation between Vitamin D and Hashimoto thyroiditis to date, the diagnosis of Hashimoto thyroiditis was generally evaluated according to TPO-Ab positivity and ultrasonographic findings. The relevance of histopathology, which is the gold standard in the diagnosis of Hashimoto thyroiditis, and serological tests is moderate. Also, the diagnostic sensitivity of diffuse heterogeneity observed on ultrasound is lower than the increased TPO-Ab level.^[11]^ In this study, we aimed to appraise whether there is a relationship between lymphocytic thyroiditis diagnosed by histopathological evaluation and vitamin D deficiency.

## Methods

Approval was obtained from the local ethics committee for this study with the decision number 3018 dated November 24, 2020. Among 1293 patients who underwent hemithyroidectomy or total thyroidectomy between 2012 and 2017, 256 patients who met the study criteria were included in the study. Their data included preoperative vitamin D analyses and histopathological examination of the thyroid specimens.

The patients with surgery in the postpartum period, with a history of lithium, amiodarone, interferon alpha, interleukin-2, and radioactive iodine therapy, malignancy other than parathyroid cancer, with hyperparathyroidism and kidney failure and/or receiving vitamin D treatment, with chronic liver disease, and epilepsy using anticonvulsants or any treatment that could affect the Vitamin D level and patients with hyperthyroidism operated for Graves’ disease, were excluded from the study. Free T3 (fT3), free T4 (fT4), thyroid-stimulating hormone (TSH), Anti-TPO, anti-thyroglobulin (TG), calcium (Ca), phosphorus (P), magnesium (Mg), alkaline, which were seen in routine preoperative blood tests phosphatase alkaline phosphatase (ALP), parathyroid hormone (PTH), 25(OH)D levels, presence of preoperative hyperthyroidism and histopathological examination of thyroid samples were evaluated.

Pathological examinations of the patients' thyroid specimens were performed by an experienced endocrine pathologist as usual. Two groups were formed considering the presence of lymphocytic thyroiditis (Group 1) and the absence of lymphocytic thyroiditis (Group 2), which were determined as the main or secondary pathology as a result of pathological examination. Vitamin D deficiency was identified as the level <20 ng/mL (50 nmol/L) and Vitamin D insufficiency were defined as the level between 21 and 29 ng/mL (525–725 nmol/L).^[12]^

The patients were divided into quartiles according to their preoperative vitamin D and TSH values. Vitamin D levels were determined as <6.21 ng/mL, 6.22–10.69 ng/mL, 10.7–22.68 ng/mL, and >22.69 ng/mL for the first, second, third, and fourth quartiles, respectively. TSH levels were determined as <0.94 uU/mL, 0.95–1.58 uU/mL, 1.59–2.4 uU/mL, and >2.4 uU/mL for the first, second, third and fourth quartiles, respectively.

### Laboratory Measurements

Serum fT3, fT4, TSH, Anti-TPO, Anti-TG, PTH, and 25(OH)D3 levels were analyzed on an automated analyzer, Beckmann Coulter DXI-800, using chemiluminescent immunoassay technique (Beckmann Coulter DXI-800, California, USA).

Ca, Mg, P, and ALP levels were measured in Beckmann Coulter AU-680 biochemistry autoanalyzer (Beckmann Coulter AU-680, California, USA). Preoperative anti-TPO level above 35 and anti-TG level above 40 were accepted as positive.

### Pathological Examinations

Pathological examinations were performed by an experienced endocrine pathologist. Lymphocytic thyroiditis was graded as follows:

1. Presence of few and scattered lymphocytes
1. Focal, dense lymphocytic infiltrates
1. Diffuse, dense lymphocytic infiltrates.

According to this grading, the morphology seen in Hashimoto’s thyroiditis was graded as diffuse dense infiltrate. The histopathological findings were characterized by lymphocytic infiltration with prominent germinal centers forming lymphoid follicles, oncocytic changes, varying degrees of fibrosis and atrophic thyroid follicles. Oncocytic cells (Hurthle cell, oxyphilic cell, and Ashkenazi cell) are large, granular cells including eosinophilic cytoplasm, large and round nuclei with prominent nucleoli, defined in the thyroid gland. They especially accompany chronic lymphocytic thyroiditis. In classical and fibrous variants of chronic lymphocytic thyroiditis, all of the thyrocytes may appear oncocytic. In addition, lymphocytic infiltration within or around the tumor tissue in various malignancies is considered as host immune response to the tumor and is not classified as lymphocytic thyroiditis.

### Statistical Analysis

Statistical analysis was performed with IBM Statistical Package for the Social Sciences (SPSS) version 25 (SPSS Inc, Chicago, IL, USA). Results are given as mean±standard deviation (SD).

In statistical analysis, number and percentage for categorical variables, and mean, SD, minimum, and maximum for numerical variables were used. In the comparison of the groups, the differences between the ratios of categorical variables were made with the Pearson’s Chi-squared test, and nonparametric comparisons were made with the Mann-Whitney U test. The relationship between chronic lymphocytic thyroiditis and other characteristics of the patients was evaluated using the Pearson’s correlation test. Statistical significance was accepted as p<0.005.

## Results

There were 108 (92 Female/16 Male) patients in Group 1, and 148 (116F/32M) patients in Group 2, and patients in group 1 were younger (p=0.053) (Table 1). Anti-TPO (p<0.001) and anti-TG (p<0.001) antibody levels were significantly higher in Group 1 as expected (Table 1). The groups were similar in terms of gender and parathyroid malignancy rate, preoperative PTH and ALP values and the presence of hyperthyroidism, and no significant difference was found between the groups according to these characteristics (Table 1). In Pearson’s correlation test, positive correlation was found between lymphocytic thyroiditis and preoperative TSH (r=0.161, p=0.010), anti-TPO (r=0.262, p=0.000), and preoperative anti-TG (r=0.171, p=0.016) values, but negative correlation was found with age (r=–0.152, p=0.015).

**Table 1.** Comparison of the characteristics of Groups 1 and 2

	**Group 1**	**Group 2**	**p**
	**(Lymphocytic thyroiditis present) n=108**	**(Lymphocytic thyroiditis absent) n=148**	
Age	44.8±13.2	48.8±13.6	0.053*
Gender F/M (n=256)	92/16	116/32	0.176**
Preop Anti-TPO (n=203) (U/mL) (Mean±SD)	166±403	18±57	<0.001*
Preop Anti-Tg (n=197) (U/mL) (Mean±SD)	259±631	80±402	<0.001*
Preop TSH (uU/mL) (Mean±SD)	2.2±2.7	1.5±1.55	0.017*
Preop fT4	1.19±0.29	1.20±0.29	0.935*
Preop fT3	3.29±0.59	3.33±0.49	0.328*
Preop PTH (pg/mL) (Mean±SD)	50.97±22.97	62.24±62.85	0.06*
Preop Ca (mg/dl) (Mean±SD)	9.5±0.48	9.55±0.49	0.489*
Preop P (mg/dl) (Mean±SD)	3.42±0.59	3.35±0.58	0.401*
Preop Mg (mg/dl) (Mean±SD)	1.92±0.18	1.94±0.28	0.643*
Preop ALP (U/L) (Mean±SD)	76.9±27.4	78.3±36.2	0.741*
Preop vit D (ng/mL) (Mean±SD)	16.6± 15.2	14±10	0.409*
Preop vit D <20 ng/mL n (%)	73 (67.6)	107 (72.3)	0.416
Preop hyperthyroidism n (%)	26 (24.1)	25 (16.9)	0.148**
Presence of papillary thyroid cancer n (%)	61 (56.5)	73 (49)	0.236**

*: Mann-Whitney U; **: Pearson’s Chi-square; (mean±SD): Mean±Standard deviation; TPO: Thyroid peroxidase; PTH: Parathyroid hormone; TG: Thyroglobulin; fT3: Free T3; fT4: free T4; TSH: Thyroid stimulating hormone; Ca: Calcium; P: Phosphorus; Mg: Magnesium; ALP: Alkaline phosphatase.

There was no significant difference between Groups 1 and 2 in terms of preoperative vitamin D levels (16.6±15.2 vs. 14±10, p=0.409) and vit D deficiency rates (67.6% vs. 72.3%, p=0.416) (Table 1). No positive significant correlation was found between lymphocytic thyroiditis and vitamin D level (r=0.104, p=0.095).

When the patients were categorized according to their preoperative Vitamin D values, the distribution ratios of the patients in Groups 1 and 2 were 25% and 25.7% in the first quartile (Vit D <6.21 ng/mL), 19.4%, and 28.4% in the second quartile (Vit D: 6.22–10.69 ng/mL), 29.6% and 21.6% in the third quartile (Vit D: 10.7–22.68 ng/mL) and 25.9% and 24.3% in the fourth quartile (Vit D >22.69 ng/mL), respectively. There was no statistically significant difference between the groups (p=0.296) (Table 2).

**Table 2.** Lymphocytic thyroiditis and its relationship with vitamin D level

	**Preop vitamin D levels**				**Total**
	**<6.21 ng/mL**	**6.22–10.69 ng/mL**	**10.7–22.68 ng/mL**	**>22.69 ng/mL**	
Lymphocytic thyroiditis (+) (Group 1)	27	21	32	28	108
	25.0%	19.4%	29.6%	25.9%	100%
Lymphocytic thyroiditis (-) (Group 2)	38	42	32	36	148
	25.7%	28.4%	21.6%	24.3%	100%
Total	65	63	64	64	256
	25.4%	24.6%	25.0%	25.0%	100%

Preoperative TSH level was significantly higher in Group 1 (p=0.017) (Table 1). The distribution ratios of the patients in Groups 1 and 2 according to TSH levels were 33.3% and 46.9% in the first quartile (0–25%, TSH <0.94 uU/mL), 13% and 15.4% in the second quartile (25–50%, TSH = 0.95–1.58 uU/mL), 19.4% and 15.4% in the third quartile (50–75%, TSH = 1.59–2.4 uU/mL) and 34.3% and 17.7% in the fourth quartile (75–100%, TSH >2.4 uU/mL), respectively, and the differences were statistically significant. The rate of patients was higher in the 4^th^ quartile in Group 1 and in the 1st quartile in Group 2 (p=0.006) (Table 3).

**Table 3.** Lymphocytic thyroiditis and its relationship with TSH level

	**Preop TSH Levels**				**Total**
**<0.94 uU/mL**	**0.95–1.58 uU/mL**	**1.59–2.4 uU/mL**	**>2.4 uU/mL**	
Lymphocytic thyroiditis (+) (Group 1)	36	14	21	37	108
	33.3%	13%	19.4%	34.3%	100%
Lymphocytic thyroiditis (-) (Group 2)	69	30	22	26	148
	46.9%	20.4%	15%	17.7%	100%
Total	105	44	43	63	256
	41.2%	17.3%	16.9%	24.7%	100%

## Discussion

It has been demonstrated that the incidence of many autoimmune diseases increased in Vitamin D deficiency.^[5]^ Although there are studies to date reporting a relationship between low Vitamin D level and lymphocytic thyroiditis, there are also studies reporting that there is no relationship, and this issue is still controversial.^[4]^

In our study, the mean Vitamin D levels (16.6±15.2 vs. 14±10, p=0.409) and Vitamin D deficiency rates (67.6% vs. 72.3%, p=0.416) were found similar between the Groups 1 and 2. No statistical difference was also found between the patients in quartiles considering their vitamin D levels (p=0.296). Additionally, no positive significant correlation was found between lymphocytic thyroiditis and vitamin D level (r=0.104, p=0.095).

As expected, anti-TG and anti-TPO auto-antibodies were significantly higher in the group of patients with lymphocytic thyroiditis (Group 1) (p<0.001 for both). Preoperative TSH levels were also higher in patients with lymphocytic thyroiditis compared to those without (p=0.017). Autoantibody increase secondary to intrathyroidal B and T lymphocyte infiltration, thyrocyte destruction, and hypofunction due to autoimmune reaction in chronic lymphocytic thyroiditis are expected results.

Goswami et al. In India,^[13]^ they found vitamin D deficiency (<25 nmol/L) in 87% of patients, similar to our study, but they could not find a correlation between vitamin D deficiency and anti-TPO antibody positivity. And they found a weak inverse correlation between serum 25(OH)D3 and anti-TPO levels.

Effraimidis et al.^[14]^ evaluated the relationship between early stage thyroid autoimmunity and Vitamin D level in 2 controlled studies in Amsterdam. According to the control group, the researchers found no relationship between early stage thyroid autoimmunity and Vitamin D deficiency neither in individuals with a genetic predisposition for autoimmune thyroid disease in the cross-sectional study, nor in individuals with new anti-TPO positivity in the longitudinal study.

In the study of D’Aurizio et al.^[6]^ comparing 100 autoimmune thyroiditis patients with a control group consisting of 126 healthy individuals, the rates of vitamin D deficiency and median 25(OH)D3 levels were found to be similar. In addition, they found similar vitamin D levels in hypothyroid and euthyroid patients with Hashimoto’s thyroiditis, and in euthyroid and hyperthyroid patients with Graves’ disease. The researchers concluded that level of vitamin D in patients with autoimmune thyroiditis was similar to the control group like the study of Effraimidis et al.^[14]^

In the study of Yasmeh et al.,^[15]^ in which they evaluated whether vitamin D deficiency was related with chronic lymphocytic thyroiditis, they checked against the data of 97 patients having lymphocytic thyroiditis with the control group consisting of 88 healthy individuals. Mean 25(OH)D3 levels were higher in female patients in the Hashimoto group compared to the control group, and similar in males. In female patients with Hashimoto's thyroiditis, the rate of patients with normal vitamin D levels was higher and the rate of Vitamin D deficiency was lower than in the control group. Vitamin D deficiency has not been detected in women. And there were no male patients with vitamin D sufficiency. In that study, no statistically significant relationship was found between 25(OH)D3 levels and anti-TG in the Hashimoto group. However, a significant positive correlation was found between 25 (OH) D3 levels and Anti-TPO antibodies only in male patients. The researchers concluded that Hashimoto’s thyroiditis was not associated with the depth of vitamin D deficiency.

In contrast to these, some other clinical studies have claimed an association between autoimmune thyroiditis and Vitamin D deficiency. Kivity et al.^[16]^ found that patients with autoimmune thyroiditis were more likely to have vitamin D deficiency and had higher anti-TPO levels than healthy adults. They examined Hashimoto’s thyroiditis, Graves’ disease, and postpartum thyroiditis in the autoimmune thyroiditis group, and they associated Vitamin D deficiency with the presence of anti-TPO and thyrocyte destruction due to dominant T lymphocyte infiltration, especially in patients with Hashimoto’s disease.

In the study conducted by Kim^[5]^ the prevalence of vitamin D deficiency (<75 nmol/l) in 369 patients with autoimmune thyroid disease was higher than those without autoimmune thyroid disease (p=0.011). In addition, when thyroid diseases were divided into groups, the rate of vitamin D deficiency in Hashimoto's thyroiditis was found to be higher than in Graves’ disease or non-autoimmune thyroid diseases (p=0.017). Patients with Hashimoto's thyroiditis with hormonal hypothyroidism had a higher prevalence of vitamin D deficiency compared with other patients with euthyroid and subclinical hypothyroid Hashimoto's thyroiditis or without autoimmune thyroiditis. And vitamin D levels were found to be lower in these patients.

In the meta-analysis of 28 studies that first evaluated the correlation between vitamin D and autoimmune thyroid diseases by Wang et al.,^[10]^ circulating concentrations of 25(OH)D3 were found to be significantly lower in patients with autoimmune thyroiditis than in the control group. However, in this meta-analysis, the lack of standardization of Vitamin D levels according to seasonal differences and the heterogeneity between the patient groups examined were stated as limitations by the researchers.

Some studies have evaluated the relationship between vitamin D and thyroid autoantibodies. Mazokopakis et al.^[17]^ found an negative correlation between serum 25(OH)D3 levels and anti-TPO levels in 218 patients with euthyroid Hashimoto’s thyroiditis. Anti-TPO antibody levels were found to be significantly higher in patients with Vitamin D insufficiency or deficiency, compared to patients with Hashimoto’s thyroiditis with normal Vitamin D levels. Following oral Vitamin D3 supplementation given to patients with vitamin D deficiency for a while, a significant decrease was detected in the control anti-TPO levels.

Chaudhary et al.^[18]^ found that anti-TPO antibody levels were highest in the group with the lowest 25(OH)D3 levels in patients with newly diagnosed autoimmune thyroiditis. The relationship between vitamin D deficiency and lymphocytic thyroiditis has often been explained by the decrease in the immunomodulatory effect of 1,25(OH)2D3.^[19]^ Although the decrease in immunomodulation alone cannot explain this relationship, sufficient evidence could not be provided because the existing studies were cross-sectional studies. It has been considered that Vitamin D deficiency might be due to the malabsorption caused by lymphocytic thyroiditis, use of corticosteroids or hyperthyroidism, resulting in a decrease in vitamin D by accelerating bone turnover.^[20]^

The main limitations of our study are that it is a retrospective study and the vitamin D level was not seasonally distributed. However, according to the studies that used serological tests which are moderately compatible in the diagnosis of Hashimoto's thyroiditis, the strongest aspect of our study is that we used histopathology, which is the gold standard in the diagnosis of Hashimoto’s thyroiditis. However, there is still a need for extensive prospective interventional studies.

## Conclusion

Vitamin D levels were similar in patients with and without lymphocytic thyroiditis. There was a positive correlation between lymphocytic thyroiditis and age, preoperative TSH level, preoperative anti-TG, and anti-TPO antibody levels, but no significant relationship was found with vitamin D level. According to our results, no relationship was found between the presence of lymphocytic thyroiditis and Vitamin D deficiency and vitamin D levels.

### Disclosures

**Ethics Committee Approval:** Approval was obtained from Sisli Hamidiye Etfal Training and Research Hospital local ethics committee for this study with the decision number 3018 dated 24.11.2020.

**Peer-review:** Externally peer-reviewed.

**Conflict of Interest:** None declared.

**Authorship Contributions:** Concept – M.K.D. ; Design – M.K.D., M.U.; Supervision – N.A, M.U.; Materials – M.B.Y.O., I.E.A.; Data Collection &/or Processing – Z.G.D. ; Analysis and/or interpretation – Z.G.D., I.E.A.; Liteature Search – M.B.Y.O., N.A.; Writing – M.K.D., Z.G.D.; Critical review – N.A, M.U.
